# Quantification of the absolute abundance of the jejunal mucosa-associated microbiota of pigs using spike-in control

**DOI:** 10.20517/mrr.2025.100

**Published:** 2026-03-18

**Authors:** Jung Yeol Sung, Hyunjun Choi, Alexa R. Gormley, Sung Woo Kim

**Affiliations:** Department of Animal Science, North Carolina State University, Raleigh, NC 27695, USA.

**Keywords:** Absolute abundance, mucosa-associated microbiota, pig, spike-in control

## Abstract

The objective was to quantify the absolute abundance of the jejunal mucosa-associated microbiota in pigs and develop a standardized spike-in control protocol. The spike-in control, containing bacteria that are Gram-negative (*Imtechella halotolerans*) and Gram-positive (*Allobacillus halotolerans*), was diluted 100 times and 10 μL (100,000 bacterial cells of each species) was added to 100 mg of porcine jejunal mucosa samples. The relative abundance of the jejunal mucosa-associated microbiota differed between the diet groups, whereas no difference was observed in their absolute abundance. In conclusion, the spike-in control containing 100,000 cells of *Imtechella halotolerans* and *Allobacillus halotolerans *can be used to quantify the absolute abundance of microbiota in 100 mg of pig jejunal mucosa.

## INTRODUCTION

Intestinal health is essential for enhancing the health of pigs, and is influenced by the intestinal microbiota^[1-3]^. The intestinal microbiota can be classified as luminal and mucosa-associated, with different compositions due to the intestinal oxygen gradient^[4,5]^. The mucosa-associated microbiota more actively influences immune responses through direct interactions with intestinal immune cells, metabolites, and opportunistic pathogens^[4,6]^.

Relative abundance is commonly utilized to compare microbial composition between different groups to test hypotheses or draw conclusions^[7-9]^. However, when interpreting relative abundance, the relative abundance of a taxon depends on that of the other taxa^[10,11]^. In contrast, absolute abundance of one taxon is independent of other taxa, potentially reducing bias^[12,13]^. Quantifying the absolute abundance allows for comparisons across different studies and overcomes limitations of individual studies^[14,15]^. One method to measure the absolute abundance is the spike-in method^[10,14,16]^. This method utilizes a spike-in control (bacterial cells or DNA), which is foreign to the microbiota composition of the target species^[16,17]^. When added to unknown samples, the absolute abundance of other microbiota can be quantified by combining their relative abundances with the known cell counts of the spike-in control, and technical bias starting from DNA extraction can be corrected^[18,19]^.

The spike-in method has been applied to quantify the absolute abundance of microbiota in human feces^[20]^, soil^[21]^, and food^[22]^. Currently, the working range of spike-in control has not been determined for the intestinal mucosa-associated microbiota of pigs. The proportion of spike-in control relative to total microbiota should be optimized because a low proportion may result in an undetectable spike-in, whereas an excessively high proportion may hinder identification of the target microbial communities^[21,23]^. The spike-in control levels used for fecal samples should not be directly applied to mucosal samples because the mucosa typically has a lower bacterial load^[24]^. Therefore, the objective was to quantify the absolute abundance of jejunal mucosa-associated microbiota in pigs and develop a standardized spike-in protocol.

## SAMPLE PREPARATION, SPIKE-IN CONTROL, AND ANALYSIS

Animal procedures were approved by the Animal Care and Use Committee (approval No.: 25-371) of North Carolina State University (Raleigh, NC, USA). Fourteen pigs (initial body weight = 6.7 ± 0.3 kg) were assigned different diets (Diet A and Diet B) meeting nutrient requirements^[25]^ using a randomized complete block design, with initial body weight and sex as blocking factors. Individually housed pigs were euthanized at 12.8 kg. Collected jejunal mucosa samples were frozen at -80 °C and stored prior to portioning into sections weighing 100 mg ± 10 mg. Sections were transferred to a 2 mL tube (Greiner Bio-One GmbH, Kremsmünster, Austria) containing 1 mL of DNA/RNA Shield (#R1100-250, Zymo Research, Irvine, CA, USA). Samples were thawed for 20 minutes, before vortexing for 20 seconds. During sample thawing, ZymoBIOMICS Spike-in Control I (#D6320, Zymo Research, Irvine, CA, USA), stored at -80 °C, was thawed and diluted 100 times using nuclease-free water (#10977015, Thermo Fisher Scientific, Waltham, MA, USA). Then, 10 μL of the 100-fold diluted spike-in control was added to each prepared sample, and this concentration was determined based on preliminary results [Supplementary Table 1]. The total number of cells and 16S copies of the spike-in control are already known [Supplementary Table 2].

## QUANTIFYING THE ABSOLUTE ABUNDANCE OF THE JEJUNAL MUCOSA-ASSOCIATED MICROBIOTA AT THE FAMILY, GENUS, AND SPECIES LEVELS

Samples were analyzed using 16S ribosomal RNA** (**rRNA**)** sequencing (Supplementary information). The known amount of added spike-in control was calculated. The total number of 16S copies derived from the spike-in control was calculated by dividing the actual spike-in control volume added (10 μL) by the standard value of 20 μL ^[26]^, accounting for the 100-fold dilution (0.1 μL). This value is then multiplied by the 16S copy number provided by Zymo Research to determine the total 16S copies of the spike-in control added per sample^[26]^. This value remained constant across all samples, assuming a consistent laboratory technique. The number of 16S copies of the spike-in control per sample was calculated for each unique spike-in control species. In this case, the values for *Imtechella halotolerans* and *Allobacillus halotolerans* were 300,000 and 700,000 16S copies, respectively. Considering that *Imtechella halotolerans* and *Allobacillus halotolerans* contain 3 and 7 16S copies per genome, respectively, this corresponds to the addition of 100,000 bacterial cells of each spike-in control species. 

Next, the total 16S copies per sample were determined using relative abundance data. Using the constant derived from the previous step and the relative abundance of the corresponding spike-in control species, the total 16S copies were calculated as^[26]^: 

**Figure:**
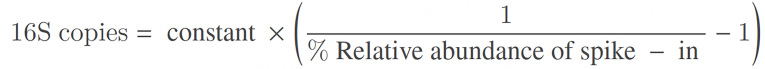


If a sample had a relative abundance of *Imtechella halotolerans* and *Allobacillus halotolerans* of 1% and 3%, respectively [Supplementary Figure 1], the 16S copies per sample can be calculated as follows^[26]^: 

**Figure:**
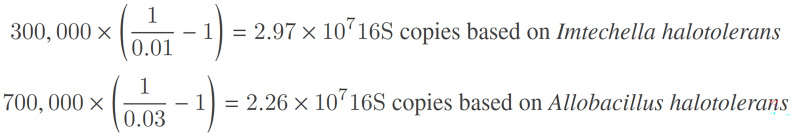


This calculation is performed for each sample, accounting for variation in the relative abundance of each spike-in control species. The resulting number of 16S copies per sample was then divided by an average number of 16S copies per bacterial genome, 5 copies, as is generally accepted for mixed bacterial populations, to estimate total cell count^[27]^. If complete genome data are available, species-specific corrections can be applied instead of assuming a mean of five copies per genome. Estimated cell counts derived from *Imtechella halotolerans* and *Allobacillus halotolerans* may differ due to variation in spike-in recovery and 16S copies per genome. Therefore, the final estimated cell count per sample may be calculated as the average of both spike-in control-based estimates^[26]^: 

**Figure:**
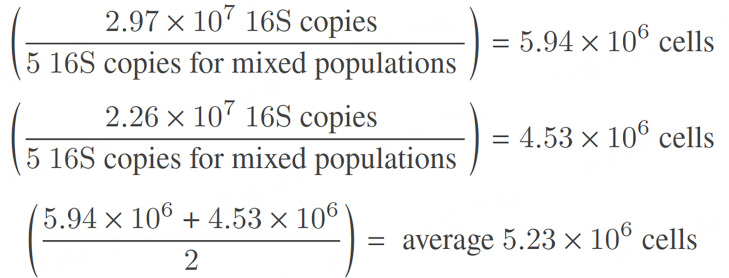


To obtain the final adjusted cell count per sample, the spike-in control cell count for each species was subtracted from the corresponding average estimated cell count. The cell count of each spike-in control species can be calculated using the relative abundance data and average estimated cell count:

**Figure:**
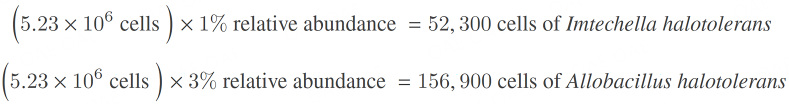


Subtracting the cell count of each spike-in control species results in a final adjusted cell count for this sample^[26]^: 

**Figure:**
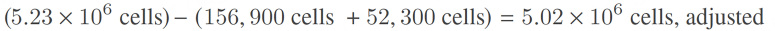


The relative abundance of the spike-in control species should also be removed from the relative abundance data and the remaining relative abundance values should be scaled to 100%. For example, the relative abundance of *Lactobacillus delbrueckii* is 45% in the initial sample, including the spike-in control species [Supplementary Figure 1]. After removal of the spike-in control species, the relative abundance of all species was multiplied by a factor of 1.042 (100 ÷ 96), accounting for the removal of the 4% relative abundance of the spike-in control. The adjusted relative abundance of *Lactobacillus delbrueckii* is 46.88% [Supplementary Figure 1]. The absolute abundance of *Lactobacillus delbrueckii* is calculated as follows^[26]^:

**Figure:**
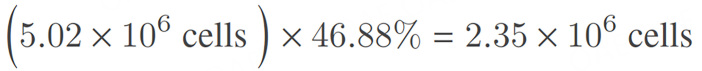


The same procedure can be applied at the family and genus levels. *Imtechella halotolerans* belongs to the genus *Lactobacillus* and the family *Lactobacillaceae*. *Allobacillus halotolerans* belongs to the genus *Allobacillus* and the family *Bacillaceae*.

## QUANTIFYING THE ABSOLUTE ABUNDANCE OF THE JEJUNAL MUCOSA-ASSOCIATED MICROBIOTA AT THE PHYLUM LEVEL

The spike-in control species will be within the same phyla as native species**;** however, their contribution can be inferred from family, genus, or species levels as relative abundance remains constant across taxonomic levels. The relative abundance of each spike-in control species should be removed from the corresponding phylum before estimating absolute abundance at the phylum level. *Imtechella halotolerans* and *Allobacillus halotolerans* are of the phyla Bacteroidota and Bacillota, respectively. For example, if a sample contained a relative abundance of 1% *Imtechella halotolerans *at the species level, its relative abundance at the phylum level would also be 1%. Therefore, if the relative abundance of Bacteroidota in the same sample was 50%, the relative abundance of 1% can be subtracted, and the relative abundance must again be scaled to 100% after removal of the spike-in control. In this case, the resulting relative abundance (49%) will also be multiplied by 1.042, for a final relative abundance of Bacteroidota at 51.06% [Supplementary Figure 2].

The relative abundance of the remaining phyla should be scaled to 100%. The corrected relative abundance of each phylum can be multiplied by the total cell count to determine absolute abundance at the phylum level. In this case, the absolute abundance of the Bacteroidota phylum is calculated as follows^[26]^: 

**Figure:**
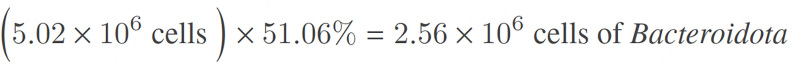


## STATISTICAL ANALYSIS

PROC MIXED in SAS 9.4 (SAS Institute, Cary, NC, USA) was used to compare the relative and absolute abundance of the jejunal mucosa-associated microbiota in pigs fed two diets (Diet A and Diet B). The statistical model was a mixed model, with diet as a fixed effect and initial body weight and sex as random effects. The significance and tendency were declared at *P* < 0.05 and 0.05 ≤ *P* < 0.10, respectively. 

## RESULTS

Fourteen jejunal mucosa samples were analyzed; more than 50,000 sequencing reads were obtained per sample, and the total number of high-quality sequences exceeded 700,000. Differences were not observed in the relative abundance of the spike-in control between dietary groups [Supplementary Table 3]. The sum of the relative abundance of the spike-in control was 4.4% in Diet A and 3.8% in Diet B. Diet B tended to increase (*P* = 0.056) the relative abundance of *Lactobacillus delbrueckii* and increased (*P *< 0.05) the relative abundance of *Lactobacillus pontis*, whereas it tended to reduce the relative abundance of *Bifidobacterium thermacidophilum* (*P* = 0.098) compared with the Diet A group [Table 1]. In contrast, differences in the absolute abundance were not observed [Table 2].

**Table 1:** Relative abundance (%) of the jejunal mucosa-associated microbiota at the species level of pigs fed two different experimental diets^1^

	**Diet A**	**Diet B**	**Pooled SEM**	***P*-value**
*Helicobacter rappini*	11.45	6.74	3.59	0.295
*Bifidobacterium dentium*	5.03	6.45	1.92	0.611
*Lactobacillus delbrueckii*	2.38	6.50	1.34	0.056
*Prevotella copri*	3.62	1.87	1.48	0.422
*Lactobacillus mucosae*	1.54	2.77	0.67	0.207
*Bifidobacterium thermacidophilum*	5.99	1.93	1.57	0.098
*Helicobacter equorum*	0.02	0.04	0.02	0.443
*Olsenella profusa*	2.54	2.87	1.77	0.830
*Lactobacillus pontis*	0.12	2.41	0.76	0.011
*Bifidobacterium boum*	0.86	2.60	0.70	0.108
*Blautia wexlerae*	0.81	1.11	0.43	0.595
*Mitsuokella multacida*	0.80	1.19	0.53	0.611
*Staphylococcus carnosus*	0.08	0.11	0.07	0.746
*Weissella cibaria*	0.28	0.19	0.28	0.736
*Lactobacillus johnsonii*	0.49	1.99	1.42	0.358
*Faecalibacterium prausnitzii*	0.47	0.38	0.16	0.712
Others	63.65	61.00	3.97	0.628

^1^Least squares mean represents 7 observations. SEM: Standard error of the mean.

**Table 2:** Absolute abundance (cells × 10^4^/ 100 mg of jejunal mucosa) of the jejunal mucosa-associated microbiota at the species level of pigs fed two different experimental diets^1^

	**Diet A**	**Diet B**	**Pooled SEM**	***P*-value**
*Helicobacter rappini*	75.2	33.3	24.8	0.260
*Bifidobacterium dentium*	25.5	37.5	12.4	0.507
*Lactobacillus delbrueckii*	39.7	64.0	28.9	0.565
*Prevotella copri*	97.8	19.8	60.6	0.375
*Lactobacillus mucosae*	21.9	19.2	15.0	0.852
*Bifidobacterium thermacidophilum*	41.6	15.7	18.6	0.267
*Helicobacter equorum*	0.7	0.3	0.5	0.633
*Olsenella profusa*	14.4	23.9	11.9	0.501
*Lactobacillus pontis*	2.6	18.5	10.4	0.110
*Bifidobacterium boum*	9.8	18.8	7.7	0.342
*Blautia wexlerae*	22.0	9.4	14.7	0.552
*Mitsuokella multacida*	13.8	11.2	8.2	0.828
*Staphylococcus carnosus*	0.7	0.4	0.4	0.688
*Weissella cibaria*	1.1	2.5	2.2	0.552
*Lactobacillus johnsonii*	17.0	14.4	17.7	0.880
*Faecalibacterium prausnitzii*	9.9	3.4	5.7	0.438
Others	750.1	474.5	323.9	0.527
Total	1146.1	769.4	470.5	0.540

^1^Least squares mean represents 7 observations. SEM: Standard error of the mean.

## DISCUSSION

Quantifying the absolute abundance enables correlation or regression analyses linking microbes with intestinal health parameters^[28,29]^. The approach can be further strengthened through meta-analysis, which overcomes the limitations of individual studies. However, quantifying the absolute abundance would be affected by variation in extraction efficiency, PCR amplification bias, 16S rRNA gene copy number heterogeneity, and taxonomic misclassification, highlighting the need for caution during analysis^[21,30]^. Because the jejunal mucosa typically has a lower bacterial load than feces^[24]^, factors such as host DNA contamination, low-biomass effects, index hopping, and reagent-derived background signals should be considered, and an appropriate spike-in control with high recovery in low-biomass environments should be selected^[31]^.

## CONCLUSION

The spike-in control containing 100,000 cells of* Imtechella halotolerans* and *Allobacillus halotolerans *can be used to quantify the absolute abundance of microbiota in 100 mg of pig jejunal mucosa. The interpretation of microbiota data can be different depending on whether relative or absolute abundance is evaluated. 

## DECLARATIONS

### Availability of data and materials

Raw sequencing data have been deposited in the National Center for Biotechnology Information (NCBI) Sequence Read Archive (SRA) under the accession number PRJNA1429549.

### Copyright

© The Author(s) 2026.
