# Supplementary material for: Quantification of the absolute abundance of the jejunal mucosa-associated microbiota of pigs using spike-in control
Source: Microbiome Res Rep. 2026 Mar 18;5(1):5. doi: 10.20517/mrr.2025.100 (PMC13091085; doi:10.20517/mrr.2025.100)
Supplement: Supplementary file 1 [file mrr-5-1-5-SupplementaryMaterials.pdf]

Supplementary Materials

Quantification of the absolute abundance of the jejunal mucosa-associated microbiota of pigs using spike-in control

Jung Yeol Sung, Hyunjun Choi, Alexa R. Gormley, Sung Woo Kim

Department of Animal Science, North Carolina State University, Raleigh, NC 27695, USA.

Correspondence to: Dr. Sung Woo Kim, Department of Animal Science, North Carolina State University, Raleigh, NC 27695, USA. E-mail: sungwoo_kim@ncsu.edu

ORCID: Sung Woo Kim (0000-0003-4591-1943)

The spike-in control used in this study passed QC test (Lot number 256655) and the bacteria in the spike-in control were quantified and matched the expected concentration. For inter batch reproducibility, the ZymoBIOMICS Microbial Community Standard (#D6300) were used as a positive control on every run, to compare across sequencing projects, and to assess agreement with the theoretical profile, which met the acceptance criteria.

DNA was extracted from jejunal mucosa samples using the ZymoBIOMICS-96 MagBead DNA Kit (Zymo Research, Irvine, CA, USA) for downstream 16S rRNA gene sequencing. Targeted sequencing preparation was carried out using the Quick-16S Primer Set V3–V4 (Zymo Research) alongside the NGS Library Preparation Kit. The primer set was designed to provide broad, unbiased coverage of bacterial 16S rRNA gene regions. Final PCR amplicons were quantified using qPCR-based fluorescence measurements and pooled at equimolar concentrations. Pooled libraries were purified using the Select-a-Size DNA Clean & Concentrator (Zymo Research) and assessed for fragment size distribution using TapeStation (Agilent Technologies, Santa Clara, CA, USA) and Qubit fluorometry (Thermo Fisher Scientific, Waltham, MA, USA).

Sequencing was performed on an Illumina NextSeq 2000 platform (Illumina, San Diego, CA, USA) using a P1 reagent kit (cat. no. 20075294; 600 cycles), with a 30% PhiX spike-in (PhiX

Control Kit V3) to increase sequence diversity and monitor run quality. Raw reads were quality filtered, denoised, and merged using the DADA2 pipeline, including trimming of low-quality bases, removal of chimeric sequences, and inference of unique amplicon sequence variants (ASVs). Sequencing depth exceeded 30,000 reads per sample after quality filtering, providing sufficient coverage for downstream microbial community analyses. Taxonomic classification was conducted using reference data from the Greengenes and SILVA databases. Any ASV data representing less than 0.5% of the total relative abundance at each level were combined together as "others". The average DNA concentration in the samples was 143 ng/μL (standard deviation = 48). The average DNA 260/280 and 260/230 ratios were 1.83 (standard deviation = 0.02) and 2.47 (standard deviation = 0.13), respectively.

Supplementary Table 1. Relative abundance (%) of the undiluted spike-in control at the species level to the jejunal mucosa-associated microbiota of pigs (pilot study)

	Undiluted spike-in control added to 100 mg of the jejunal mucosa-associated microbiota, μL				
Species	0	1	5	10	100
Imtechella halotolerans	0	19.4	23.9	27.2	36.4
Allobacillus halotolerans	0	31.2	39.5	38.6	56.8

Supplementary Table 2. Microbial composition of bacteria based on 20 μL of the undiluted spike-in control added[1]

Species	Cell count	16S copies	16S copy number per cell/genome
Imtechella halotolerans	2×10^7	6.0×10^7	3
Allobacillus halotolerans	2×10^7	1.4×10^8	7

[1]#D6320, Zymo Research, Irvine, CA, USA.

This table is adapted from the instruction manual provided by Zymo Research [1].

Supplementary Table 3. Relative abundance (%) of the 100-fold diluted spike-in control at the species level to the jejunal mucosa-associated microbiota of pigs fed two different experimental diets[1]

	Diet A	Diet B	Pooled SEM	*P*-value
Imtechella halotolerans	2.1	1.7	0.6	0.593

Allobacillus halotolerans	2.3	2.1	0.7	0.773

[1]Least squares mean represents 7 observations. SEM: Standard error of the mean.

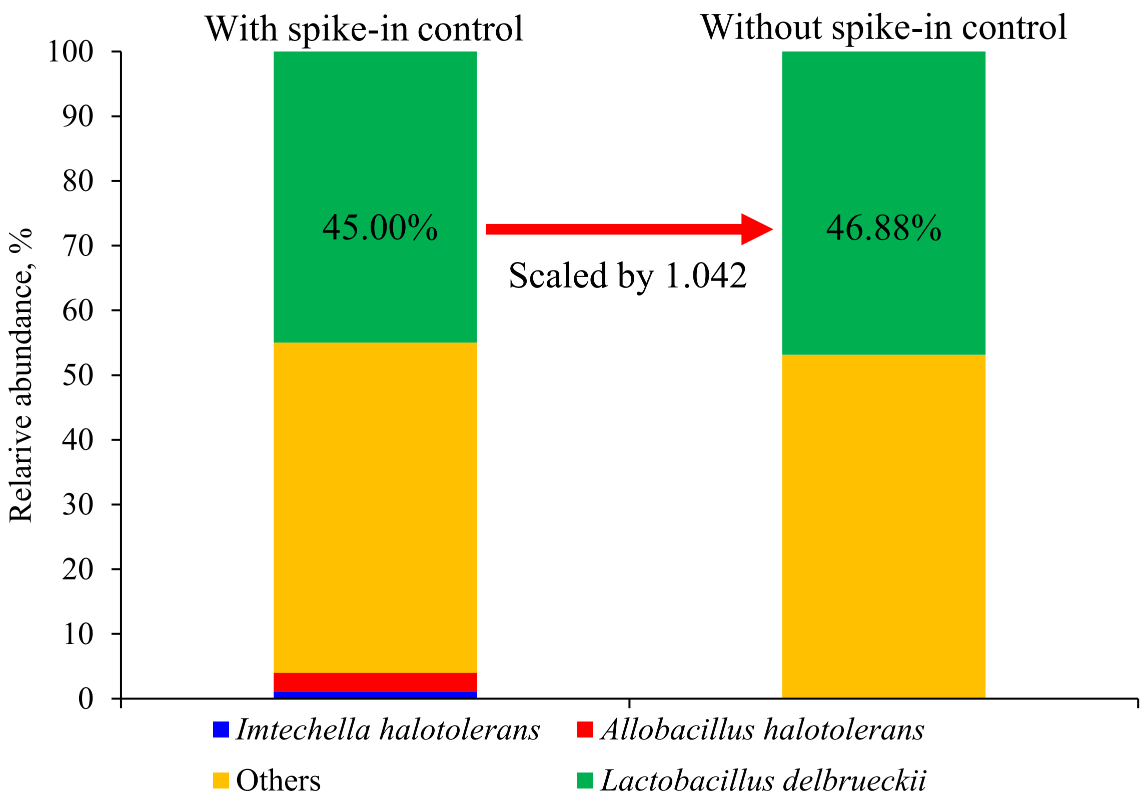

Supplementary Figure 1. Conceptual illustration of the relative abundance of the jejunal mucosa-associated microbiota with (left bar) or without the spike-in control (right bar) at the species level. Red bar (*Allobacillus halotolerans*; relative abundance: 3%) and blue bar (*Imtechella halotolerans*; relative abundance: 1%) represent the spike-in control. When the spike-in control is removed, the relative abundance of *Lactobacillus delbrueckii* is scaled by 1.042 (100 ÷ 96).

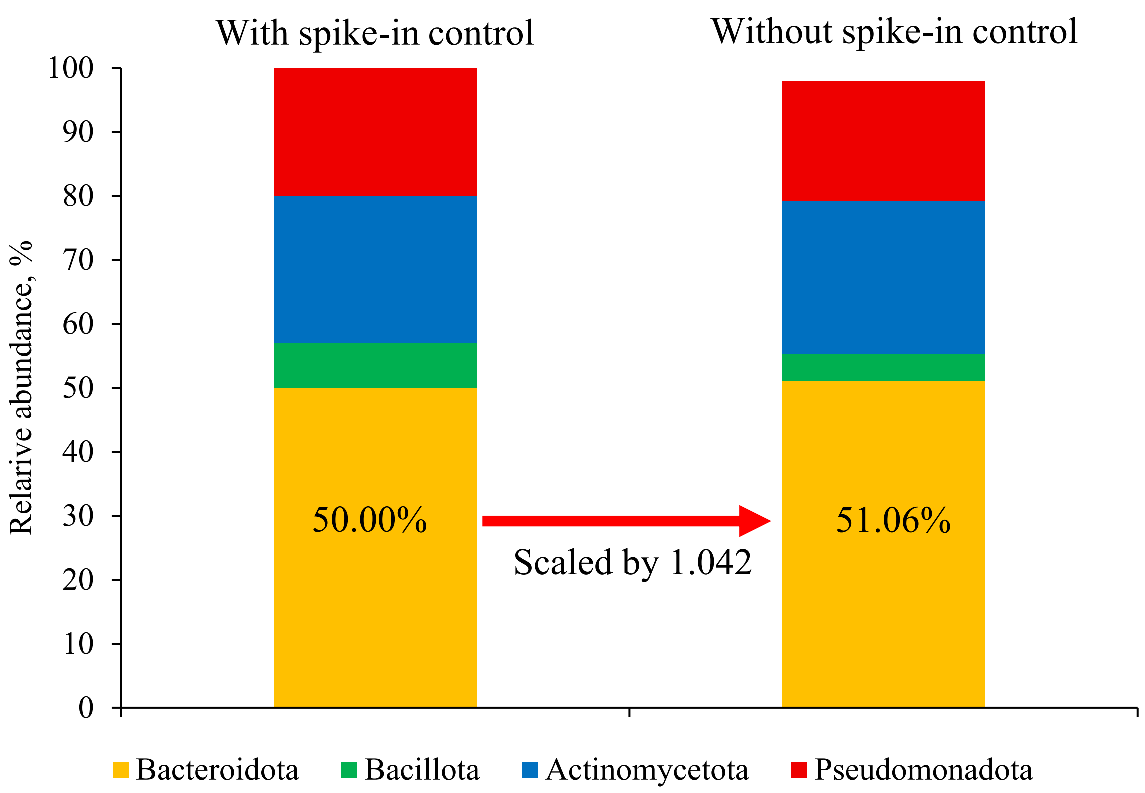

Supplementary Figure 2. Conceptual illustration of the relative abundance of the jejunal mucosa-associated microbiota with (left bar) or without the spike-in control (right bar) at the phylum level. The orange bar represents Bacteroidota shared with the spike-in control (*Imtechella halotolerans*; relative abundance: 1%) and the green bar represents Bacillota shared with the spike-in control (*Allobacillus halotolerans*; relative abundance: 3%). In this example, 1% of *Imtechella halotolerans* and 3% of *Allobacillus halotolerans* were removed from their respective phyla (Bacteroidota and Bacillota) and the relative abundance is scaled prior to evaluation of the absolute abundance. Therefore, the relative abundance of Bacteroidota without the spike-in control is 51.06% [(50% – 1%) × (100% ÷ (100% - 1% - 3%))]. Note, the other spike-in control species (*Allobacillus halotolerans*) belongs to a different phylum (Bacillota) and therefore the relative abundance of Bacillota has similarly been adjusted, in this figure.

REFERENCES

1. Zymo Research. Instruction Manual: ZymoBIOMICS™ Spike-in Control I (High Microbial Load). Ver. 1.1.5.
